# Rhesus Monkey Rhadinovirus Uses Eph Family Receptors for Entry into B Cells and Endothelial Cells but Not Fibroblasts

**DOI:** 10.1371/journal.ppat.1003360

**Published:** 2013-05-16

**Authors:** Alexander S. Hahn, Ronald C. Desrosiers

**Affiliations:** New England Primate Research Center, Harvard Medical School, Southborough, Massachusetts, United States of America; University of Southern California, United States of America

## Abstract

Cellular Ephrin receptor tyrosine kinases (Ephrin receptors, Ephs) were found to interact efficiently with the gH/gL glycoprotein complex of the rhesus monkey rhadinovirus (RRV). Since EphA2 was recently identified as a receptor for the Kaposi's sarcoma-associated herpesvirus (KSHV) (Hahn et al., Nature Medicine 2012), we analyzed RRV and KSHV in parallel with respect to Eph-binding and Eph-dependent entry. Ten of the 14 Eph proteins, including both A- and B-type, interacted with RRV gH/gL. Two RRV strains with markedly different gH/gL sequences exhibited similar but slightly different binding patterns to Ephs. gH/gL of KSHV displayed high affinity towards EphA2 but substantially weaker binding to only a few other Ephs of the A-type. Productive entry of RRV 26-95 into B cells and into endothelial cells was essentially completely dependent upon Ephs since expression of a GFP reporter cassette from recombinant virus could be blocked to greater than 95% by soluble Eph decoys using these cells. In contrast, entry of RRV into fibroblasts and epithelial cells was independent of Ephs by these same criteria. Even high concentrations and mixtures of soluble Eph decoys were not able to reduce by any appreciable extent the number of fibroblasts and epithelial cells productively entered by RRV. Thus, RRV is similar to its close relative KSHV in the use of Eph family receptors for productive entry into B cells and endothelial cells. However, RRV uses a separate, distinct, Eph-independent pathway for productive entry into fibroblasts and epithelial cells. Whether KSHV also uses an Eph-independent pathway in some circumstances or to some extent remains to be determined.

## Author Summary

Here we show that the gH/gL glycoprotein complex of rhesus monkey rhadinovirus binds to and mediates entry of virus into target cells via cellular Ephrin receptor tyrosine kinase proteins. Rhesus monkey rhadinovirus is a gamma-2 herpesvirus that is a close homolog of the human Kaposi's sarcoma-associated herpesvirus (KSHV; HHV-8). While KSHV uses EphA2 principally or exclusively for entry, RRV is able to use a broader range of both A-type and B-type Eph receptors. The use of Eph proteins as receptors is conserved despite substantial sequence variation in gH/gL between two RRV strain types and between RRV and KSHV. Importantly, while entry of RRV into B cells and endothelial cells was completely dependent on the Eph receptors by a variety of criteria, entry of RRV into fibroblasts and epithelial cells was essentially independent of Eph receptors by these same criteria. Thus, RRV uses a separate, distinct, Eph-independent pathway for productive entry into fibroblasts and epithelial cells. Whether KSHV also uses an Eph-independent pathway in some circumstances or to some extent remains to be determined.

## Introduction

The gamma-2 herpesviruses, also called rhadinoviruses, are a distinct subfamily of the lymphotropic herpesviruses. The rhesus monkey rhadinovirus (RRV) is a natural infectious agent found at high frequency in both captive and feral populations of rhesus monkeys (*Macaca mulatta*) [1]. RRV is a rhesus monkey homolog of the human Kaposi's sarcoma-associated herpesvirus (KSHV, human herpesvirus 8, HHV-8). RRV and KSHV share a nearly identical genome organization, high gene-for-gene sequence similarity, and an identical array of captured host genes [2], [3]. Unlike KSHV, RRV can be grown lytically and to high titer on monolayer cells, principally early passage primary rhesus fibroblasts. Both RRV and KSHV establish persistent infection of B cells *in vivo* [4], [5] and of established B cell lines [6], [7]. The B cell appears to be the principal site of persistence of both RRV and KSHV *in vivo* in their natural hosts [4], [5], [8]. RRV has been associated with B cell malignancies similar to those caused by KSHV [9]–[11]. While RRV-positive retroperitoneal fibromatosis has been observed in animals inoculated with RRV strain 17577 [10], no tight association of RRV with solid tumors has been reported.

Cellular integrins, either alpha3beta1 [12] or alphaVbeta3 [13], have been reported to serve as receptors for mediating entry of KSHV into target cells. The KSHV interaction with integrins is mediated by glycoprotein B and it is not known whether other viral glycoproteins may participate in the integrin-mediated entry process. Interestingly, neither the RGD-sequence in gB nor binding of gB to integrin alpha3beta1 or alphaVbeta3 is conserved between KSHV and RRV [13]. DC-SIGN has also been reported to function as a receptor for KSHV on activated B cells [14]. However, interaction of DC-SIGN with a specific viral glycoprotein has not been demonstrated and, as with HIV-1 [15], DC-SIGN may be simply serving as an adhesion molecule on the surface of the cells to bring virions into close proximity to the entry-mediating receptors. In 2007, Kaleeba et al. reported that the cysteine transporter xCT serves as a receptor for KSHV glycoprotein-mediated membrane fusion [16]. While function of xCT in KSHV glycoprotein-mediated fusion has been demonstrated, additional details on whether xCT is a ‘classical’ receptor that directly binds to a KSHV glycoprotein or rather a critical host factor for the fusion process are so far not available. Another report by Veettil et al. claims that xCT interacts with integrins in a time-dependent manner upon KSHV infection and affects viral gene expression rather than fusion and entry [17]. xCT, like other members of the SLC7 amino acid transporter family, dimerizes with a heavy chain 4F2hc/CD98 [18]. These dimers of CD98 with a light chain from the SLC7 family form the 4F2 antigen [19] which is also known as fusion regulatory protein 1 (FRP-1) [20] and appears to be involved in a multitude of different membrane fusion processes [20]–[22]. This supports a role for xCT in fusion, but perhaps not as a classical receptor whose engagement triggers the viral fusion protein. More recently, Hahn et al. reported that an Ephrin receptor tyrosine kinase, EphA2, serves as a cellular entry receptor for KSHV [23]. EphA2 binds with high avidity to the gH/gL glycoprotein complex of KSHV and several different approaches of interfering with this receptor interaction inhibited entry of KSHV into a variety of different target cells.

Here, we describe the conserved use of Ephrin receptor tyrosine kinases (Ephs) for entry of the related rhadinovirus RRV into specific target cells; entry of RRV into B cells and endothelial cells could be blocked by more than 95% with soluble Eph decoys. By the same means, cell-cell transmission of KSHV into a B cell line could be abrogated. These results provide support for the conserved use of Eph by both KSHV and RRV for entry into B cells and endothelial cells. However, our results indicate that RRV can also utilize a separate, distinct entry pathway that is independent of Ephs for productive entry into fibroblasts and epithelial cells. This Eph-independent pathway is apparently not available to any appreciable extent to RRV in B cells and endothelial cells.

## Results

### Identification of Ephs as interaction partners for RRV gH/gL complexes

**Figure 1:**
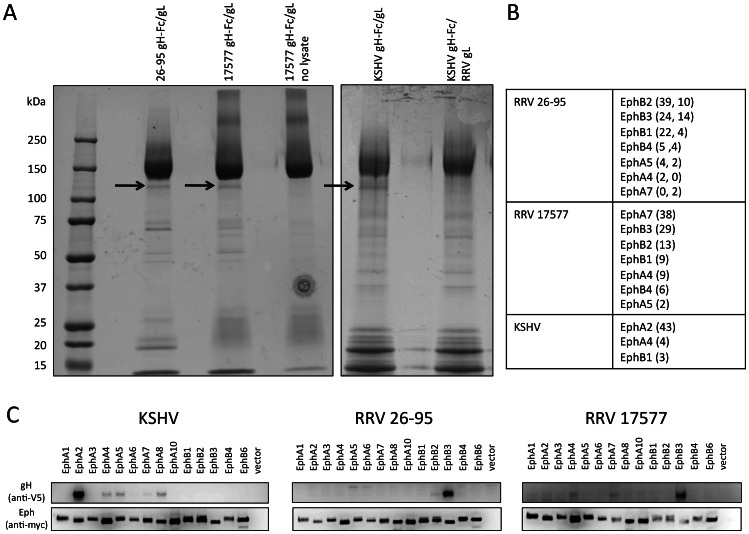
Identification of cellular interaction partners of RRV gH/gL. (**A**) Purification of proteins interacting with the ectodomains of RRV 26-95, RRV 17577 and KSHV. Lysates of 293T cells were incubated with gH-Fc/gL (Strep epitope-tagged) complexes from RRV 26-95 or 17577 pre-coupled to Streptactin beads. After elution with desthiobiotin, the eluate was re-precipitated with Protein A sepharose, washed, and subjected to gel electrophoresis followed by colloidal coomassie staining. Arrows indicate Eph-containing bands. (**B**) Summary of proteins identified in the indicated band with the respective gH/gL complexes. The numbers of peptides identified for each protein in one sample are given in brackets. Where more than one number is given, several samples were analyzed and the first number is from the first sample, the second from the second sample. (**C**) Pairwise immunoprecipitation of individual Eph proteins with gH/gL of KSHV, RRV 26-95 and RRV 17577. Full length Eph proteins (myc epitope-tagged) were recombinantly expressed in 293T cells. The lysates were normalized for equal expression with the lysate of non-transfected 293T cells. The recombinant Eph proteins were immobilized with myc antibody on Protein G beads in a first round of immunoprecipitation. The immobilized proteins were then incubated with equal amounts of lysates from 293T cells transfected with expression constructs for the respective gH (V5 epitope-tagged)/gL complexes, followed by washing and Western Blot analysis.

To identify cellular proteins that associate with the extracellular domain of gH/gL complexes of RRV in an unbiased way, we performed large-scale, two-step immunoprecipitation. RRV 26-95 and RRV 17577 have highly divergent gH and gL sequences [24]; therefore, gH and gL from both isolates were included. After a first pulldown of proteins from cell lysates from 293T cells with gH-Fc/gL complexes from RRV 26-95, RRV 17577 or KSHV immobilized on Streptactin, the complexes were specifically eluted with desthiobiotin. Subsequently, the protein complexes were re-precipitated from the eluate with Protein A to eliminate background and then subjected to gel electrophoresis and staining with colloidal coomassie. Examples of the stained gels are shown in Fig. 1A. Co-precipitating proteins were excised from the gel and identified by tryptic digest, LC-MS and peptide sequencing. KSHV gH-Fc/gL was included in our experiments as a reference (Fig. 1A rightmost three lanes), with a mixed dimer between KSHV gH-Fc and RRV 26-95 gL as an additional control. Proteins of an apparent molecular weight of approximately 110 kDa were found to precipitate with gH/gL complexes of both RRV strains and were identified as a mixture of Ephs (Fig. 1B). As previously reported [23], KSHV gH/gL precipitated predominantly with EphA2 (Fig. 1B).

### Specificity of RRV and KSHV gH/gL complexes for Ephs

**Figure 2:**
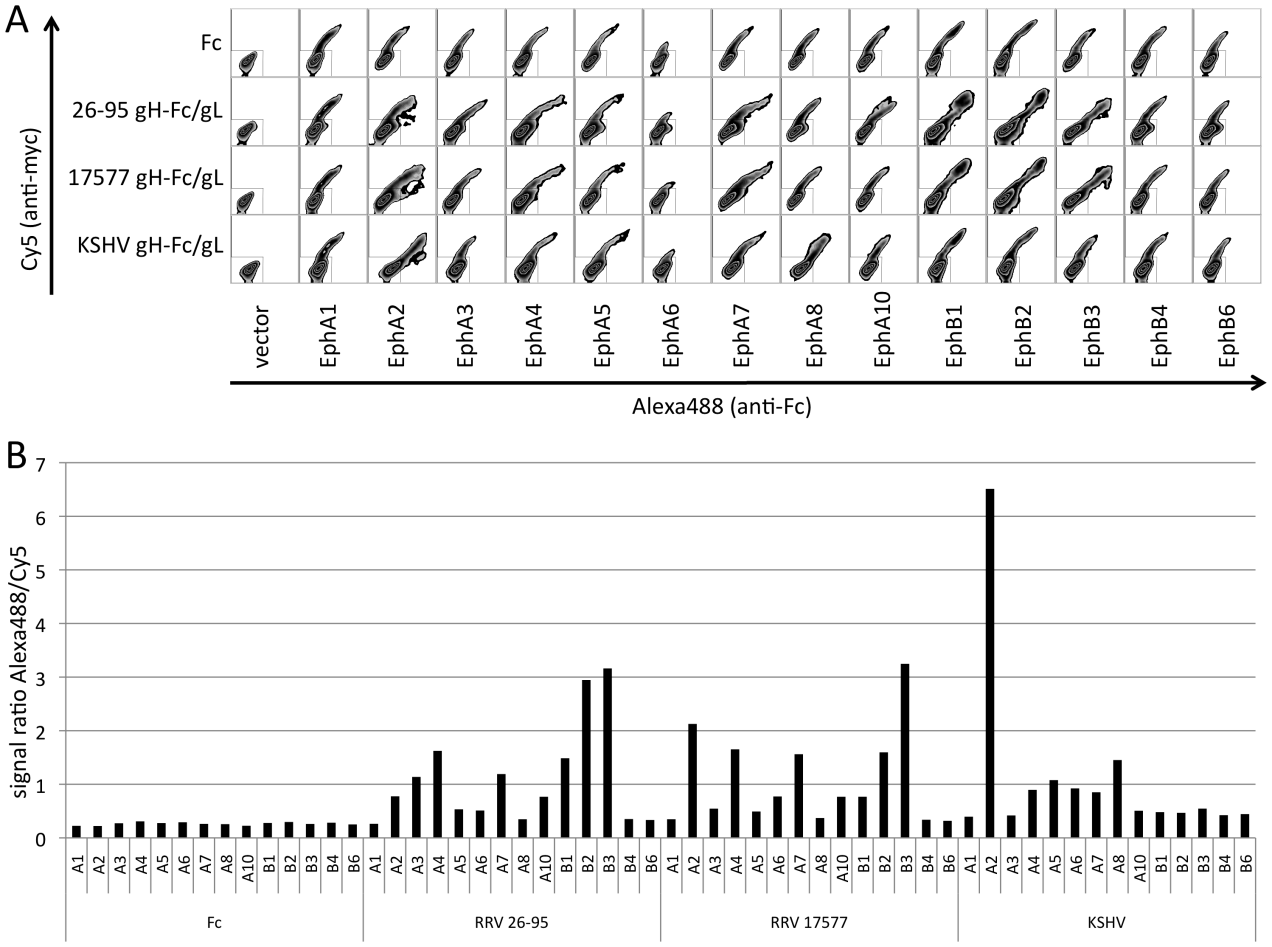
Interaction of rhadinoviral gH/gL complexes with Eph family receptors. (**A**) Binding of soluble gH-Fc/gL complexes to Eph family proteins. 293T cells were transfected with the indicated myc epitope-tagged Eph constructs. The cells were fixed and permeabilized, and both Eph expression and binding of soluble gH-Fc/gL complexes from RRV 26-95, RRV 17577 or KSHV at 10 nM was assayed by flow cytometry. Fc was used as a control. (**B**) As a semi-quantitative gauge of binding, the ratio of the geometric mean fluorescence intensities for Eph-expression and gH-Fc/gL binding was calculated. The whole area of the dot blot containing cells positive for Cy5 and/or Alexa488 except for the box gate containing negative cells in the lower left corner was analyzed, and the ratio of the geometric mean of the fluorescence intensity (MFI) for bound gH-Fc/gL (Alexa488) over the MFI for Eph expression (Cy5) was calculated. Values for the Fc control protein are shown to the left and represent the assay background.

As a first approach of verifying the above results, we performed pairwise co-immunoprecipitations with each of the three gH/gL complexes and each of the 14 Ephs expressed as full length proteins with a C-terminal myc epitope tag (Fig. 1C). We found both RRV gH/gL complexes to interact prominently with EphB3 and KSHV gH/gL to interact with EphA2 under the conditions used in these immunoprecipitation experiments. These conditions appeared to eliminate some interactions with Ephs other than EphB3 that were detected with the gH/gL complexes of both RRV strains in the initial mass spectrometry experiments. To achieve a wider dynamic range and to better compare binding of the rhadinoviral gH/gL complexes to different Ephs, 293T cells were transfected with expression plasmids for the 14 known human Ephs and binding of the individual gH/gL complexes to the transfected cells was assayed by flow cytometry (Fig. 2A). The cells were stained for both the Eph constructs via their C-terminal myc tag and the gH-Fc/gL complexes via the Fc portion. The ratio of the geometric mean of the fluorescence of the bound gH-Fc/gL complex (Alexa488 via secondary antibody to Fc-portion) divided by the geometric mean of the fluorescence of the detected Ephs (Cy5 via myc-tag) was calculated as a semi-quantitative gauge for binding (Fig. 2B, Suppl. Fig. S1). The uniformly low and practically identical ratios for binding of the Fc control protein (Fig. 2B, left) for each Eph protein validated this approach. The RRV gH/gL complexes of both RRVs were able to bind to both A and B type Ephs, slightly favoring the B-type EphB3. KSHV on the other hand bound EphA2 most efficiently of all gH/gL-Eph pairs and was found to interact with other A-type Ephs only weakly, and not with B-type Ephs. Interestingly, all the gH/gL complexes shunned EphB4 completely, with no detectable binding in both assays.

### Relevance of Eph-driven entry for RRV infection of primary cells and cell lines of different lineages

**Figure 3:**
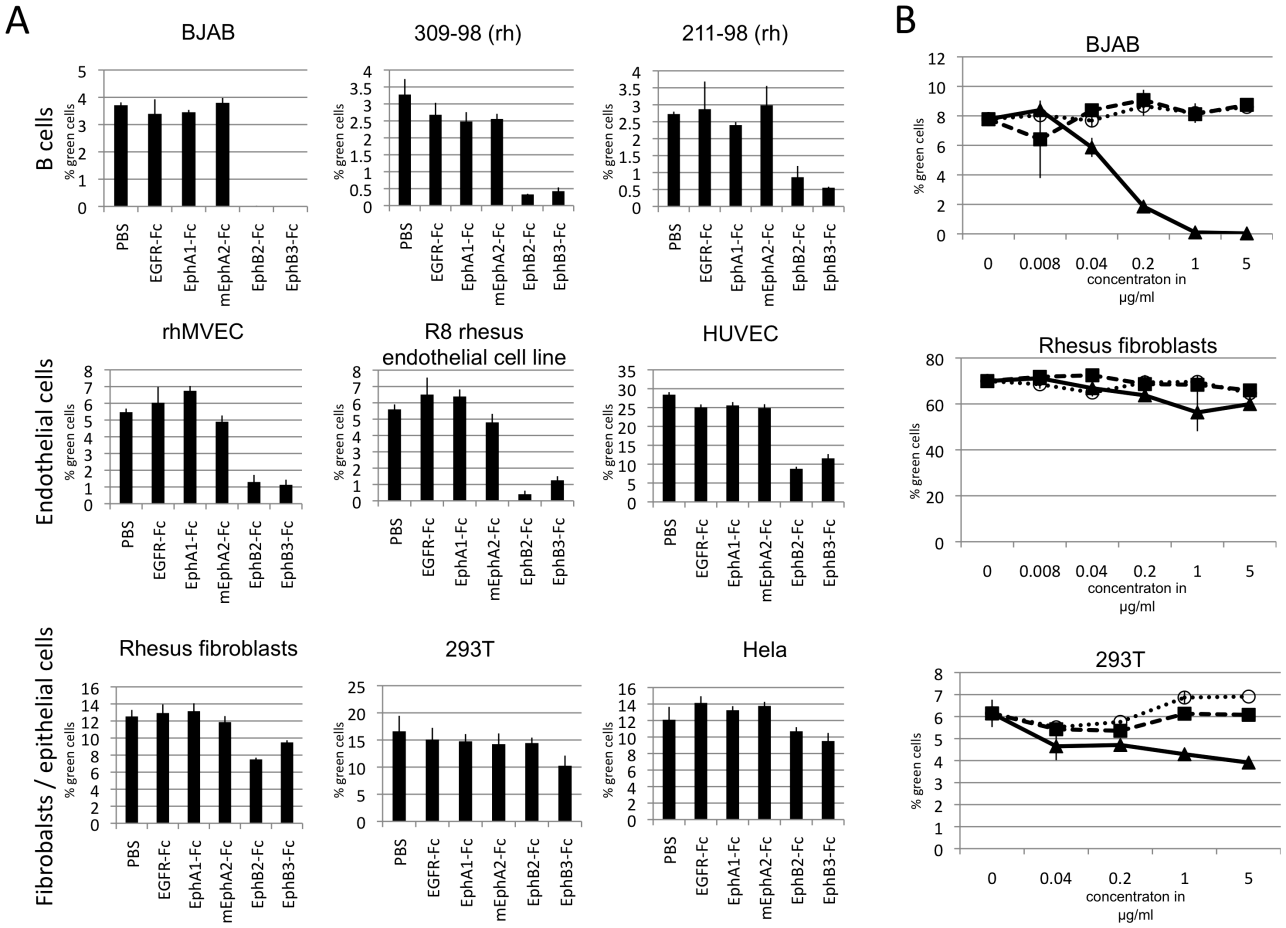
Inhibition of RRV entry by soluble Eph proteins. (**A**) Inhibition of RRV entry into cells of different lineages through block with soluble Eph proteins. RRV-GFP 26-95 was pre-incubated with EphA1-Fc, mEphA2-Fc, EphB2-Fc, EphB3-Fc or EGFR-Fc as a control prior to infection. All proteins were used at 1 µg/ml. (n = 4 for rhMVEC, R8 and HUVEC, else n = 3; error bars indicate sd) (**B**) Dose dependent inhibition of RRV entry by soluble Eph proteins. BJAB, rhesus fibroblasts and 293T cells were infected with RRV-GFP 26-95 that was pre-incubated at the indicated concentrations prior to infection with either murine EphA2-Fc (black boxes), EphB3-Fc (black triangles) or EGFR-Fc (open circles) as control. Viral entry as indicated by GFP expression was quantified by flow cytometry. (n = 3 per data point for 293T and BJAB, n = 2 for rhesus fibroblasts; error bars indicate sd for n = 3 or range for n = 2).

To examine the actual usage of Ephs as receptors for RRV entry, blocking experiments were performed. Cell lines and primary cells derived from B cells, endothelial cells, fibroblasts and epithelial cells were infected with RRV-GFP 26-95 which was pre-incubated with soluble Eph-Fc fusion proteins to compete with the cellular Ephs for binding to the viral gH/gL complex. Eph-Fc fusion proteins (R&D Systems) were used as decoy receptors, all but EphA2-Fc of human origin. Murine EphA2-Fc was used in order to utilize identically prepared proteins from the same supplier since human EphA2-Fc was not available. Murine and human EphA2 are highly homologous (92% amino acid identity, Suppl. Table S1, both function as KSHV receptor [23], and soluble forms of both molecules block KSHV entry, see below). As RRV efficiently enters cells of both human and rhesus origin, we chose a selection of cells available to us from both species and infected those cells with RRV-GFP 26-95 reporter virus after pre-incubation of the virus with the soluble Ephs as decoys. We found that infection of the epithelial cell lines 293T and Hela as well as infection of primary rhesus fibroblasts was at most marginally affected by block with soluble Ephs (inhibition <50% vs EGFR-Fc control, Fig. 3A, lower panel). In stark contrast, entry of RRV 26-95 into both B cells and endothelial cells was heavily impaired or even abolished by soluble Ephs at 1 µg/ml (Fig. 3A, upper and mid panels). In dose-inhibition curves (Fig. 3B), it became evident that soluble Ephs abolish RRV 26-95 entry into BJAB B cells already at low concentrations, whereas entry into 293T and rhesus fibroblasts was affected only marginally and inhibition did not increase with increasing concentrations of the soluble Ephs.

**Figure 4:**
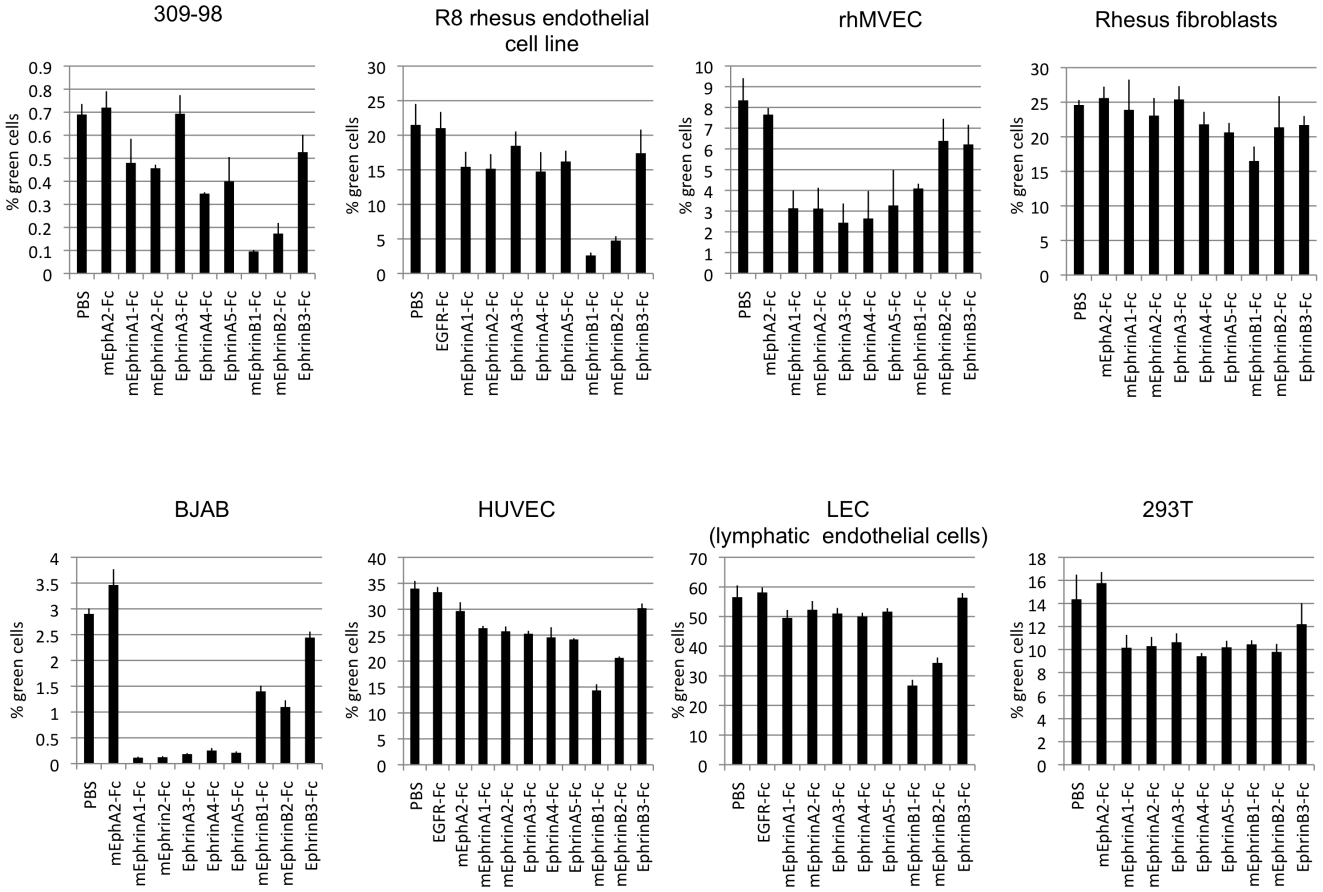
Block of RRV entry by soluble Ephrins. Primary cells and cell lines were incubated with the soluble Ephrin-Fc fusion proteins at 5 µg/ml for 30 minutes prior to infection with GFP encoding RRV 26-95. Entry was quantified by flow cytometry. EGFR-Fc or mEphA2-Fc were used as controls in addition to PBS. (n = 4 for rhMVEC , LEC, and R8, else n = 3; error bars indicate sd).

A similar picture was obtained by blocking entry of RRV 26-95 into several cell lines by pre-incubation of the cells with soluble Ephrin-Fc proteins (Fig. 4). Ephrins are the natural ligands of the Eph receptors. The name ‘Ephrin’ is itself an abbreviation derived from ‘Eph family receptor interacting protein’ [25]. Soluble Ephrin-Fc ligands may block access to the Ephs by both interfering with binding and perhaps also by triggering endocytosis, thus removing the Ephs from the cell surface. There are eight Ephrins, classified into A- and B-type. A-type Ephrins are anchored in the plasma membrane through a GPI-anchor, the B-type Ephrins are classical type I transmembrane proteins [25], [26]. This classification also corresponds to the ligand specificities, with A-type Ephrins binding preferentially A-type Ephs and B-type Ephrins binding B-type Ephs [27], although some cross-binding has been reported [28]. The actual numbers of the Ephs and Ephrins are not indicative of their specificities (Suppl. Fig. S2). A commercially available panel of soluble human and murine Ephrin-Fc fusion proteins (fused to human IgG1 Fc) covering all Ephs with their specificities was used; murine Ephrins bind human receptors and vice versa and the specificities of single Ephrins usually overlap several receptors with different affinities (according to manufacturer, Suppl. Fig. S2). Entry of RRV 26-95 into the two B cell lines (Fig. 4, BJAB and 309-98, leftmost panels) was strongly inhibited by Ephrin ligands. Entry into rhesus microvascular endothelial cells (rhMVEC, Fig. 4 top, 3^rd^ panel) was also inhibited but to a lesser extent. Entry into those cells was less affected by any one single Ephrin, but to a noticeable extent by several different Ephrins indicating involvement of both A- and B-type Ephs. These findings mirror the broad specificity for Eph-binding of RRV gH/gL described above (Figs. 1 and 2). The profile of effective Ephrins clearly varied from one cell to another, likely reflecting usage of different Ephs on different cells. For example, entry of RRV 26-95 into BJAB (B cell derived) was blocked most effectively by A-type Ephrins, which indicates A-type receptor usage. On the other hand, entry into 309-98 (B cell derived) cells and R8 (endothelial) was blocked most effectively by B-type Ephrins, which indicates B-type receptor usage.

### Relevance of Eph engagement for KSHV entry into adherent cells and a B cell line

**Figure 5:**
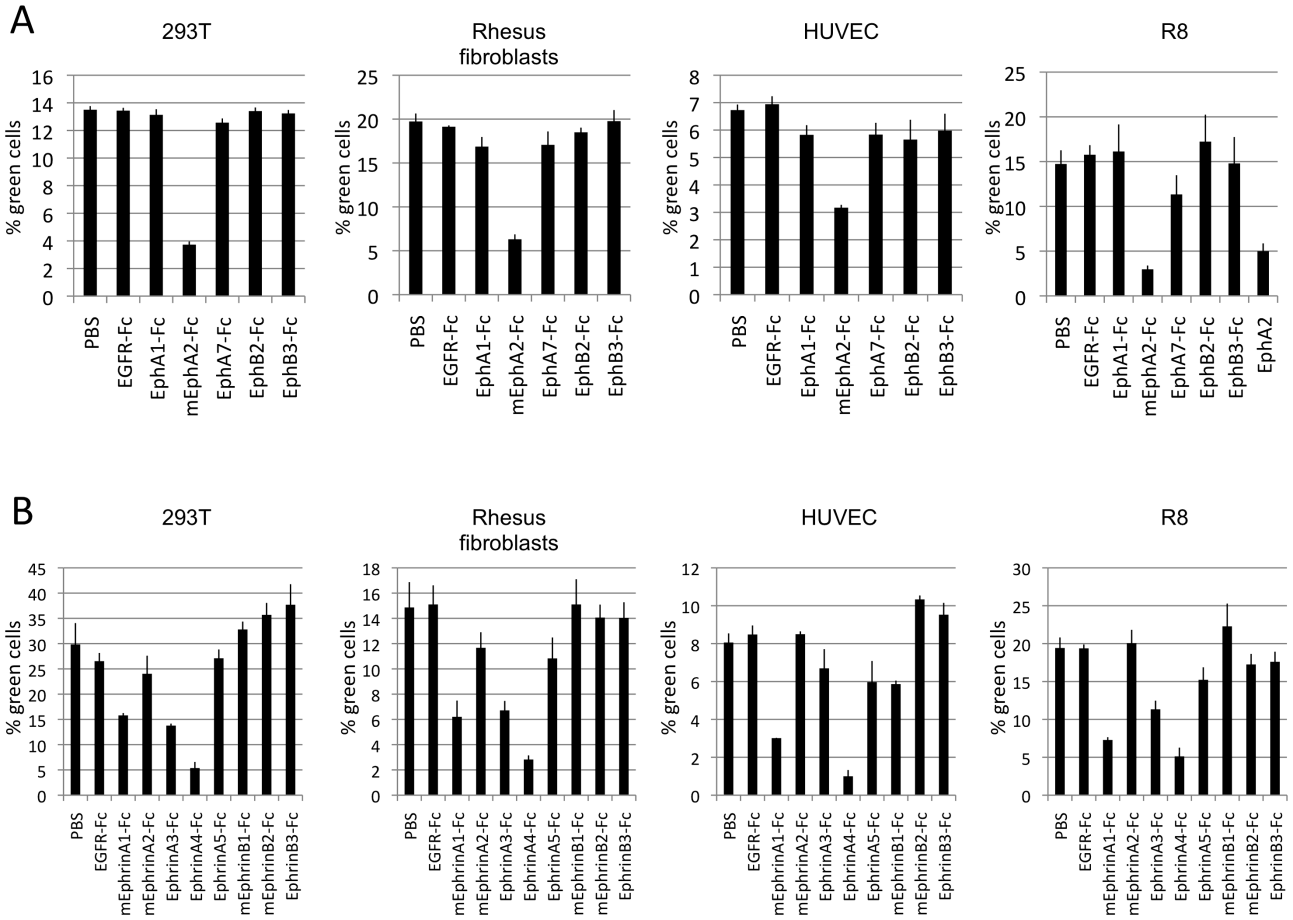
Inhibition of KSHV entry by soluble Eph proteins and soluble Ephrins. (**A**) rKSHV.219 was pre-incubated with the indicated soluble Eph-Fc fusion proteins at 1 µg/ml for 45 min. Entry was quantified by flow cytometry. (n = 3; error bars indicate sd) (**B**) Inhibition of KSHV entry by soluble Ephrins. Target cells were pre-incubated with the indicated Ephrin-Fc fusion proteins at 5 µg/ml for 30 min followed by infection with rKSHV.219. Entry was quantified as in (A). PBS and EGFR-Fc serve as controls. (n = 4 for HUVEC and R8, else n = 3; error bars indicate sd).

Completely analogous to our experiments with RRV 26-95, KSHV was pre-incubated with soluble Ephs before infection of 293T cells, rhesus fibroblasts, human umbilical vein endothelial cells (HUVEC), and R8 rhesus endothelial cells. Only soluble mEphA2-Fc but not EphA1-Fc, EphA7-Fc, EphB2-Fc or EphB3-Fc was able to block entry of KSHV into those cells at 1 µg/ml (Fig. 5A). Recombinant human EphA2 (without Fc part) performed comparably to recombinant murine EphA2-Fc protein (Fig. 5A rightmost panel). Blocking with soluble Ephrins at 5 µg/ml affected KSHV infection only when the cells were incubated with A-type Ephrins, and with EphrinA4-Fc having the greatest effect (Fig. 5B). This fits with EphA2 as the principal receptor when compared to the binding properties of the individual Ephrin-Fc constructs used (Suppl. Fig. S2). EphA2 binds efficiently to the Ephrin A1, A3 and A4 Fc-fusion proteins that were effective in our blocking assays. While EphA2 and EphA4 both strongly bind to EphrinA4-Fc, EphA4 also avidly binds to EphrinA5-Fc, which was without a pronounced effect in our blocking assays and is bound very weakly by EphA2. Moreover, EphrinA1-Fc and EphrinA3-Fc were also active in our KSHV blocking assay but do not interact as avidly with EphA4 as does the inactive EphrinA5-Fc. Thus, EphA2 binds with high avidity to all Ephrins that were active in our blocking assay, but only weaker to those that were inactive, while the ligand preferences of EphA1, EphA4, EphA5 and EphA7 do not match the blocking activities of the Ephrins towards KSHV.

**Figure 6:**
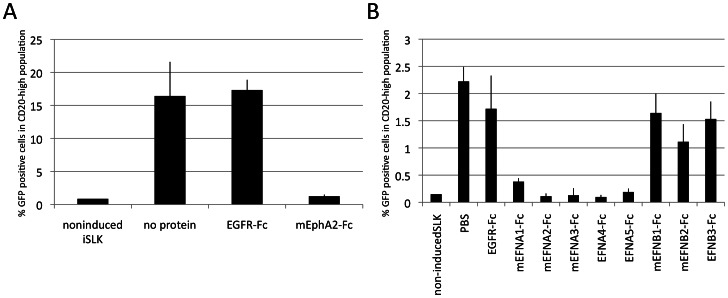
Inhibition of KSHV cell-cell transmission into a B cell line by soluble Eph proteins and soluble Ephrins. BJAB B cells were co-cultured for 10 days (**A**) or 4 days (**B**) with lytically induced iSLK.219 cells in the presence of the indicated proteins at 5 µg/ml. The cells were then harvested and analyzed for expression of the GFP reporter gene by flow cytometry. BJAB cells were distinguished from iSLK.219 cells by a two-step gating strategy, first selecting by FSC/SSC for the B cell population and then by gating for CD20 expression. The percentage of green CD20-positive cells from co-culture with non-induced iSLK cells represents the assay background. PBS and EGFR-Fc serve as controls. (n = 3; error bars indicate sd).

Infection of B cells with KSHV proved to be technically more difficult than infection with RRV 26-95, but was achieved by co-culturing BJAB B cells with lytically induced iSLK.219, as recently described by Myoung et al. [7]. Co-culture was performed in the presence of soluble Ephs, Ephrins or a control protein. The CD20-positive population representing the B cells was analyzed for GFP expression. Entry into BJAB was again inhibited by soluble EphA2-Fc (Fig. 6A) and A-type Ephrins (Fig. 6B). Surprisingly, in this setting all A-type Ephrins blocked entry to background levels. Blockage by both EphA2 decoy receptor and Ephrin ligands at 5 µg/ml resulted in virtually complete inhibition of entry as opposed to the results with adherent cells, where always a certain residual level of entry was observed.

### Cell specific use of Ephs by RRV

**Figure 7:**
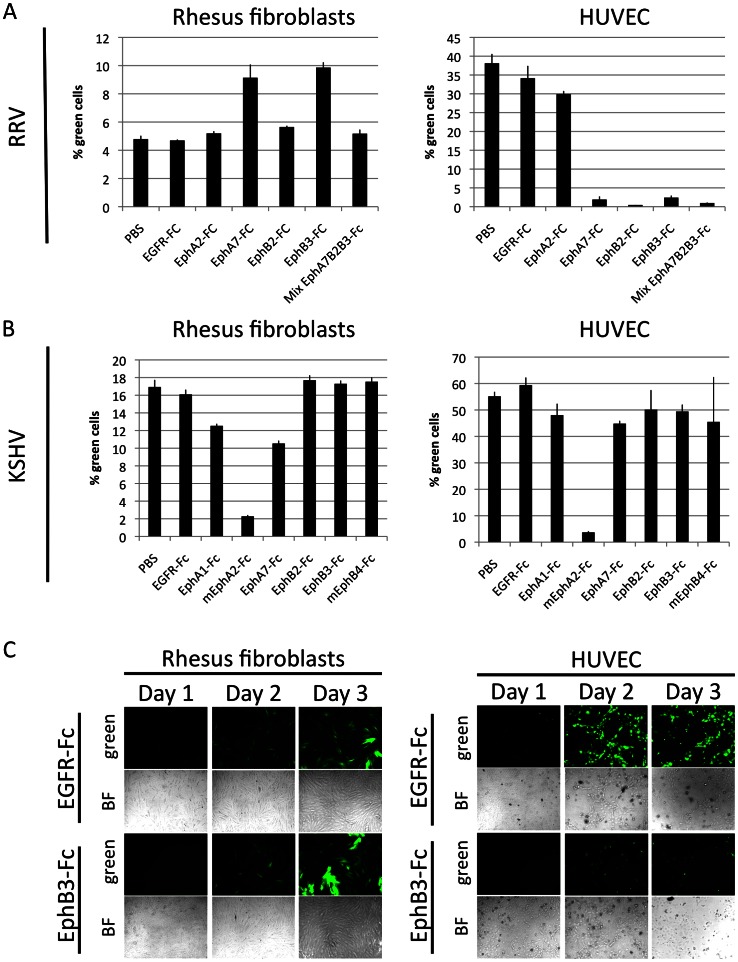
Differential entry of KSHV and RRV into fibroblasts and endothelial cells. (**A**) RRV-GFP 26-95 was pre-incubated with the indicated Eph-Fc fusion proteins at 20 µg/ml or a mix of three at 6.66 µg/ml. The virus was then divided and inoculated onto primary rhesus fibroblasts or HUVEC cells. Entry was quantified by flow cytometry. (n = 3; error bars indicate sd) (**B**) KSHV was pre-incubated with the indicated Eph-Fc fusion proteins at 20 µg/ml prior to infection of rhesus fibroblasts or HUVEC and entry was quantified as above. (n = 3; error bars indicate sd) (**C**) HUVEC and rhesus fibroblasts were infected with RRV-GFP 26-95 in the presence of either EphB3-Fc or EGFR-Fc (control) at 10 µg/ml. Pictures were taken on day 1, 2 and 3 after infection (BF: brightfield, green: GFP).

When directly compared, a striking difference between endothelial cells and fibroblasts with regard to infection with RRV 26-95 became obvious (Figs. 3 and 4). There was no or only very little effect of Eph blocking agents on RRV 26-95 entry into fibroblasts, with minor variations between no or little effect between experiments. It could be argued that the high functional MOI close to 1 achieved on early passage fibroblasts (Fig. 3B) might mask inhibition because the assay has already reached its upper saturation limit. We thus used a higher passage, less permissive batch of fibroblasts (lower functional MOI with same virus) for another experiment, and compared highly permissive HUVEC cells head-to-head. HUVEC were chosen because of all endothelial cells tested, HUVEC were the ones with the apparently smallest effect of Eph decoy receptor blocking (Fig. 3A). Again, RRV 26-95 entered rhesus fibroblasts apparently unaffected by block with soluble Ephs or combinations of soluble Ephs even at 20 µg/ml (Fig. 7A and 7C, left). We do not know the significance of the slight increase in entry into fibroblasts with two of the Eph-Fc proteins at high concentrations in Fig. 7A. In stark contrast to the result with fibroblasts, when the same Eph-pre-incubated RRV inoculum was used on HUVEC in parallel, RRV infection was greatly diminished (Fig. 7A, right). Block with the RRV interacting Ephs (EphA7, EphB2, EphB3) as soluble decoys inhibited entry of RRV into HUVEC almost completely (Fig. 7A and 7C, right). In contrast to RRV, KSHV entry into both fibroblasts and HUVEC was inhibited by mEphA2-Fc (Fig. 7B) to a similar degree. Using twenty times the concentration of soluble decoy receptor as compared to Fig. 5, marginal inhibition of KSHV entry was also observed with EphA1-Fc (−22%) and with EphA7-Fc (−35%) on rhesus fibroblasts; this could reflect weak interaction of those two Ephs with KSHV gH/gL. Obviously, KSHV and RRV differ with respect to entry into fibroblasts, but not into endothelial cells. The extreme discrepancy between RRV entry into rhesus fibroblasts and into HUVEC was clearly visible from day one through three post infection, not only with the low-permissive fibroblasts used in Fig. 7A but also with primary fibroblast that exhibit a functional MOI comparable to HUVEC (Fig. 7C).

We next investigated the influence of MOI on degree of inhibition using a high-titered RRV-YFP 26-95. A 1∶20 dilution of this stock infected 95% of HUVEC cells, indicating an MOI substantially greater than 1 at this dilution on those cells (Suppl. Fig. S3A). We used both numbers of YFP-positive cells (Suppl. Fig. S3A) and mean fluorescence intensity (Suppl. Fig. S3B) as the readouts and we used 10 µg/ml of EphB3-Fc for blocking (Suppl. Figs. S3A and S3B). At the highest levels of input virus, inhibition based on total number of YFP-positive HUVEC dropped from over 90% to as low as 65% (Fig. S3C). However, based on mean YFP fluorescence intensity, which is not saturated at high MOI, the degree of inhibition was over 95% even at the highest MOI on HUVEC cells (Suppl. Figs. S3B and S3D). Although some inhibition of entry into fibroblasts by EphB3-Fc was observed in this experiment, the extent of inhibition was much less than with HUVEC. In a total of four experiments with high concentrations of soluble Eph decoy receptors so far, we have seen low levels of inhibition in fibroblasts in two of them (Fig. 3A and Suppl. Fig. S3A) and no inhibition in two others (Fig. 3B and Fig. 7A), likely reflecting variation in batch and passage history of these primary cells. Nonetheless, even when measuring at earlier timepoints which is slightly more sensitive towards detecting inhibition, reduction of entry as determined by the number of positive cells is limited to 50% maximum in fibroblasts as compared to 99% in HUVEC and does not respond to increasing concentrations of the blocking agent (Suppl. Fig. S3E).

**Figure 8:**
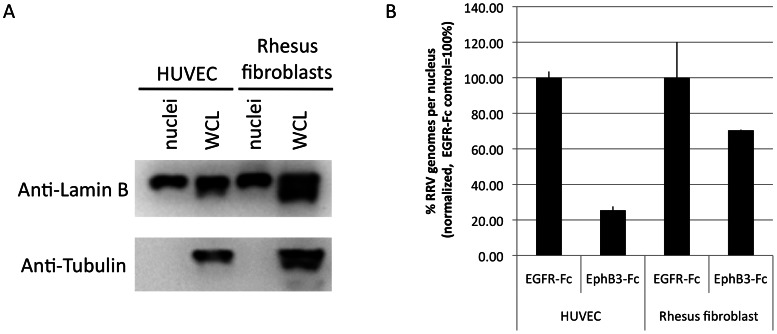
Nuclear delivery of RRV into HUVEC is Eph-dependent. (**A**) Western Blot analysis of nuclear fractions (nuclei) and whole cellular lysates (WCL) prepared from HUVEC and rhesus fibroblasts. Whole cellular lysate and nucleic fraction were probed for lamin B (nuclear marker) and tubulin (cytoskeleton). (**B**) Quantification of RRV genomes in the nuclear fraction 4 h post infection. HUVEC or rhesus fibroblasts were infected with RRV for 4 h. Virus was pre-incubated with EphB3-Fc or EGFR-Fc (control) for 45 minutes. The cells were washed, harvested by trypsinization, and nuclei were prepared. Total DNA was extracted from the nuclei and copy number of the viral genome and the cellular GAPDH locus was quantified by realtime PCR. The ratio of RRV/GAPDH copy number for the control infection (EGFR-Fc) in each cell type was set to 100%. The average of three independent experiments is shown, error bars represent the standard deviation. The difference in HUVEC is significant (p = 0.00003), the difference in rhesus fibroblasts is not (p = 0.125) (n = 3, two sided student's t-test with unequal variance).

We next asked whether the dependence on Ephs for productive entry of RRV into HUVEC cells as measured by reporter gene expression is reflected by levels of incoming virion DNA reaching the nucleus. Nuclei were prepared from infected cells at four hours post infection in a confluent monolayer six well plate under conditions that yield 70–95% (MOI>1) infected cells. The quality of the separation protocol was controlled by Western Blot analysis on the nuclear marker lamin B and the cytoplasmic/cytoskeletal marker tubulin (Fig. 8A). Measurement of the number of RRV genomes in the nuclear fraction of fibroblasts and HUVEC by quantitative realtime PCR confirmed our previous results in that EphB3-Fc soluble decoy receptor indeed inhibited the accumulation of virion DNA in the nucleus. Nuclear accumulation of the viral genome in HUVEC four hours post infection was significantly reduced by 74% in the presence of 5 µg/ml EphB3-Fc (Fig. 8B), whereas nuclear delivery to fibroblasts may have been reduced slightly but the difference was not significant.

### Importance of vesicle acidification for Eph-mediated entry

**Figure 9:**
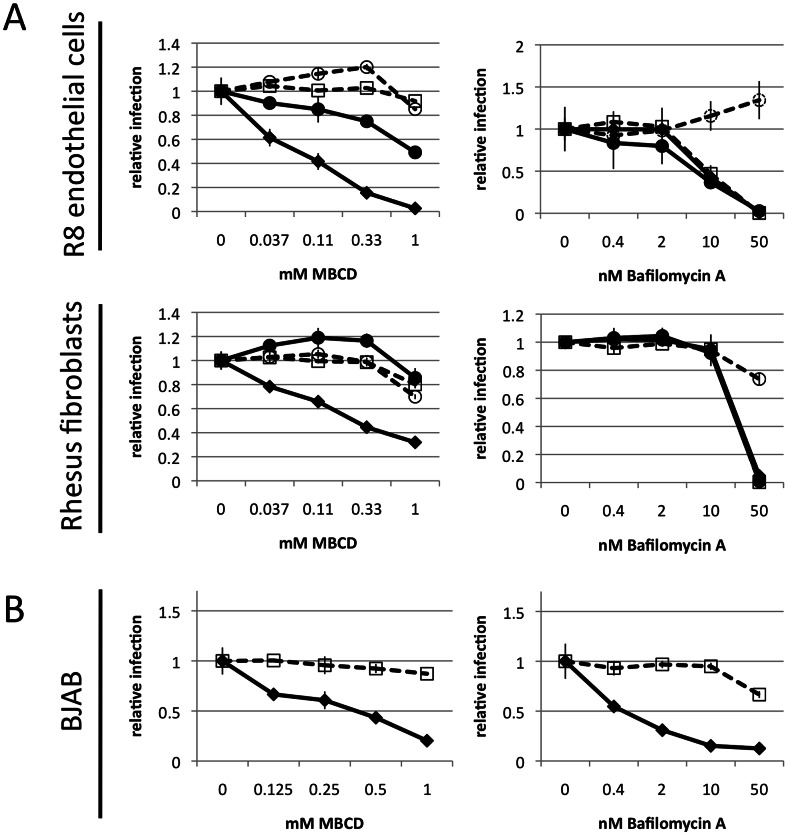
Rhadinovirus entry is dependent on vesicle acidification. (**A**) R8 endothelial cells or rhesus fibroblasts were pre-incubated for 1 h with MBCD or bafilomycin A at the indicated concentrations. The cells were then inoculated with rKSHV.219 (black line, circles) or RRV-GFP 26-95 (black line, diamonds). SIVmac329deltanef-GFP pseudotyped with A-MLV (dashed line, open circles) or VSV-G (dashed line, open boxes) envelope proteins was used in parallel as controls. Entry was assayed by GFP expression after 48 h. (**B**) The same assay as in (A) was performed with BJAB B cells with RRV-GFP 26-95 (black diamonds). An exclusion dye based cell viability assay (dashed line, open boxes) was included to control for toxicity. (n = 3; error bars indicate sd).

A number of reports implicate endocytosis followed by vesicle acidification as the main mechanism of entry for KSHV [29]–[31] and RRV [32]. Likewise, lipid rafts were reported to play a major role in KSHV entry [33]. We thus examined the Eph-dependent entry pathway of RRV 26-95 into R8 endothelial cells and the largely Eph-independent entry pathway into rhesus fibroblasts with respect to sensitivity towards inhibition of vesicle acidification and lipid raft formation. KSHV, which enters both cell types principally in an Eph-dependent fashion, was analyzed in parallel. Bafilomycin A, a highly specific inhibitor of the vacuolar ATPase, or methylbetacyclodextrin, a cholesterol depleting agent that destroys lipid rich microdomains [34], were chosen for inhibition of vesicle acidification and lipid raft formation, respectively. In addition, VSV-G and A-MLV pseudotyped lentiviruses encoding GFP were included as controls for the specificity of bafilomycin A on vesicle acidification and cell viability. VSV-G mediated fusion is pH dependent whereas A-MLV mediated fusion is not [35]. We found that inhibition of vesicle acidification strongly interfered with entry of RRV and KSHV into both R8 endothelial cells and rhesus fibroblasts (Fig. 9A, right panels) and also with entry of free RRV 26-95 into a B cell line (Fig. 9B, right panel). The dose-response inhibition curves obtained for RRV and KSHV in the presence of bafilomycin A were virtually indistinguishable from curves obtained with a VSV-G pseudotyped lentivirus, while a A-MLV pseudotyped lentivirus was unaffected (Fig. 9A, right panels). Cholesterol depletion by MBCD yielded a slightly more nuanced picture. We found cholesterol depletion by MBCD to effectively inhibit entry of RRV 26-95 into all cells tested (Fig. 9A and 9B, left panels). KSHV on the other hand was only moderately affected by MBCD on R8 endothelial cells, and not at all on rhesus fibroblasts.

## Discussion

There are common features and there are also differences when comparing KSHV and RRV for productive entry into target cells. The principal common feature is the recognition and use of Eph receptors by viral gH/gL in the entry process. Productive entry could be blocked by soluble forms of specific Ephs when used as blocking agents (Figs. 3 and 5A), and soluble forms of specific Ephrins (Figs. 4 and 5B), i.e. the ligands for the Eph receptors, were also able to specifically inhibit productive viral entry. Both KSHV and RRV appear to use receptor-mediated endocytosis for the productive entry process (Figs. 9A and 9B). Interestingly, entry of RRV and KSHV was always sensitive to inhibition of vesicle acidification. In contrast, only entry of RRV was generally sensitive to cholesterol depletion. Entry of KSHV into R8 endothelial cells was only marginally affected and entry into rhesus fibroblasts was not sensitive to cholesterol depletion (Fig. 9A, left panels). This implicates low pH as a common feature in entry, but not the use of lipid rafts, at least not on all cell types. An interesting question here would be whether cell-specific localization of certain Eph receptors in lipid rafts governs this behavior. At least for some cells, EphA2 was described to be lipid raft associated [36].

RRV gH/gL appears to recognize a broader range of Eph receptors than gH/gL of KSHV, and the highest affinity ‘preferred’ receptor differs between the two viruses: EphA2 for KSHV and EphB3 for RRV. Inhibition by the soluble Eph receptor decoys mirrors this preferred affinity; RRV was blocked by soluble EphB3 but not EphA2 whereas KSHV was blocked by soluble EphA2 but not EphB3. The ability of RRV strain 26-95 and RRV strain 17577 to target a similar spectrum of Ephs (Fig. 2) is quite remarkable given the impressive divergence in sequences in the external domain of gH and in gL [24]. The biggest difference between the two seems to be that strain 17577 exhibits a much higher affinity towards EphA2. It remains to be determined whether the fact that EphB4 is not bound by all three viruses has biological correlates. Interestingly, EphB4 is a marker for venous endothelium [37] and is downregulated through a phenotypic switch from venous to arterial endothelium in KS [38].

The blocking experiments with different Ephrin ligands (Fig. 4) clearly demonstrate that RRV is capable of not only binding but also using both A- and B-type Ephs for entry. KSHV on the other hand relies heavily on EphA2, and the spectrum of Ephrins with blocking activity clearly implicates EphA2 (Fig. 5B), which also fits the binding profile of KSHV gH/gL (Figs. 1 and 2) and previous observations [23], [36]. Only cell-cell transmission of KSHV into BJAB was also sensitive to inhibition with a broader spectrum of A-type Ephrins, but not B-type Ephrins (Fig. 6). The explanation for this broader spectrum with cell-cell transmission of KSHV in BJAB cells is not clear at the present time.

Our data argue strongly that Eph family receptors are serving as classic receptors for receptor-mediated entry of RRV into target B and endothelial cells, rather than some facilitating function such as cellular activation. The efficiency with which productive entry and delivery of the viral genome to the nucleus (Fig. 8B) could be blocked by Eph-specific reagents and the specificity of the effects are the strongest arguments to support this contention. Productive entry could be routinely blocked to >90%, even by relatively low concentrations of soluble EphB3 receptor (Fig. 3) and by soluble forms of the natural ligands (Ephrins) (Fig. 4). The inability of these same reagents (soluble EphB2 and EphB3 decoy receptors) to appreciably impact productive entry of KSHV (only susceptible to EphA2 decoy receptor) into the very same cells or of the same RRV stock into fibroblasts and epithelial cells argues not only for the specificity of the effect but also for the use of Ephs as receptors for receptor-mediated entry into B and endothelial cells.

Our data show convincingly that RRV achieves productive entry into fibroblasts and epithelial cells independent of Eph receptor usage (Fig. 2B, Figs. 7A and 7C, Fig. 8B, Suppl. Fig. S3) and, therefore, must be using different mediators for entry into these cells. While Ephs are expressed on those cells, the Eph-independent route of entry is predominant.

There is precedent in the herpesvirus family for different modes of entry for the same virus into different types of cells. In the case of EBV, different gH/gL complexes are used to infect lymphocytes and epithelial cells. EBV gH/gL associates with gp42 for the infection of B cells through type II HLA molecules [39]; for epithelial cells, EBV gH/gL binds directly to integrins to trigger fusion [40]. In the case of cytomegalovirus, a pentameric complex of gH/gL/UL128/UL130/131 is used for the infection of epithelial and endothelial cells [41], but the accessory glycoproteins UL128-131 are dispensable for infection of fibroblasts [42].

Based on the precedent of these other herpesviruses, it is logical to speculate that gH/gL of RRV may similarly complex with another envelope protein to mediate entry into fibroblasts and epithelial cells. It is also possible that entry of RRV into fibroblasts and epithelial cells may be independent of gH and/or gL. Along these lines, MHV-68 deleted in the gL locus has been shown to be replication competent [43]. The gamma-2 herpesviruses encode fewer glycoproteins than other subfamilies of herpesviruses and thus are convenient for the investigation of these alternate modes for viral entry. Furthermore, the difference between RRV's dependence on Ephs for entry into B and endothelial cells and its independence of Ephs for entry into fibroblasts and epithelial cells is dramatic. Whatever receptor is being used by RRV for entry into fibroblasts and epithelial cells, our results predict its absence or lack of function in B cells and endothelial cells.

Does KSHV differ from RRV in terms of its ability to use an Eph-independent route for entry? Certainly, productive entry of KSHV into all of these same cell types is significantly blocked by soluble EphA2 receptor decoys or soluble Ephrins, and sometimes even abrogated (Figs. 5 and 6), [23]. However, there is a certain residual low level of infection in many cell types that can't be readily blocked by interfering with Eph receptor function, also noticed in a previous study [23]. More work will be needed with KSHV to clarify this issue.

With regard to the virus-associated malignancies, Ephs clearly are the relevant receptor family. Our data demonstrate that Ephs are not only crucial for infection with free virus, but also for cell-cell transmission of KSHV to a B cell line (Fig. 6). Entry of both RRV and KSHV into endothelial cells and B cell lines was dramatically inhibited by all ways of interfering with Eph receptor-engagement. From a wider perspective, it is fascinating that both tumor viruses use receptors whose expression or function is massively deregulated in tumors [44]. It is tempting to speculate what additional roles Eph receptors may play in rhadinovirus-associated tumorigenesis, also with respect to the differences in the receptor specificities of KSHV and RRV, and whether these differences contribute to the ability of KSHV to cause Kaposi's sarcoma.

## Materials and Methods

### Cell culture and transfections

293T cells (ATCC), Hela cells (ATCC) and primary rhesus fibroblasts (NEPRC, Harvard Medical School) were cultured in DMEM supplemented with 10% fetal bovine serum (FBS) (Invitrogen), 25 mM HEPES (Invitrogen), 100 Units/ml Penicillin (Invitrogen), 100 µg/ml Streptomycin (Invitrogen) and 2 mM Glutamine (Invitrogen). BJAB (a kind gift from Michaela Gack, NEPRC, Harvard Medical School), 309-98 and 211-98 rhesus B cell lines (both a kind gift from Fred Wang, Harvard Medical School) were kept in RPMI supplemented with 20% FBS, 25 mM HEPES, 100 Units/ml Penicillin, 100 µg/ml Streptomycin, 1 mM sodium pyruvate (Invitrogen) and 2 mM Glutamine. All endothelial cells were cultured in EGM-2 endothelial growth medium (Lonza) and culture vessels were pre-coated with Attachment Factor (Invitrogen). Human umbilical vein endothelial cells (HUVEC) cells were purchased from Lonza. Lymphatic endothelial cells (LEC) from juvenile donors were purchased from Promocell. R8 telomerase immortalized rhesus endothelial cells and primary rhesus microvascular endothelial cells were a kind gift from Jay Nelson (Oregon Health & Science University).

Large scale transfection of 293T cells for protein production was performed using the Mammalian Cell Profection Calcium Phosphate Kit (Promega) with 20 µg DNA per 10 cm cell culture dish. Small scale transfection for FACS analysis was performed using LipofectamineLTX and Plus reagent (Invitrogen) according to the manufacturer's protocol with a DNA(µg):Plus(µl):LipofectamineLTX(µl) ratio of 1∶1∶1.

### Plasmids

All Eph coding sequences were cloned into the pcDNA4amychis (Invitrogen) backbone vector via appropriate restriction enzymes. cDNAs were obtained by RT-PCR from 293T cells and from commercial sources where necessary and sequences are representative of the NCBI reference sequence if not stated otherwise (Suppl. Table S1). Sequence similarity with orthologs was determined using the BLAST algorithm [45]. The EphB6 construct has a conservative amino acid exchange E998D. Viral gH and gL cDNAs were cloned into the pcDNA6aV5His (Invitrogen) backbone. Sequence identity was verified by DNA sequencing from both ends. The sequence of RRV 26-95 gH and gL was codon-optimized for expression as described elsewhere [46]. The soluble ectodomains of gH from RRV 26-95 (amino acids 21-697) and RRV 17577 (amino acids 19-694) without signal peptide were inserted behind a heterologous signal peptide of murine IgG-kappa into pAB61Strep in a fashion analogous to KSHV gH/gL [47], resulting in C-terminally fused IgG1 Fc-fusion proteins (gH-Fc) with a tandem Strep-Tag at the C-terminus.

### Recombinant proteins

Proteins bearing a tandem Strep-Tag at their C-terminus were purified from 293T cell culture supernatant. 293T cells were transfected by calcium phosphate transfection with expression plasmids based on the pAB61Strep vector described earlier [47]. The protein-containing cell culture supernatant was first filtered through 0.22 µm PES membranes (Millipore) and then passed over 0.5 ml of a Streptactin (Qiagen) matrix in a gravity flow Omniprep column (BioRad). Bound protein was washed with 50 ml phosphate buffered saline pH 7.4 (PBS) (Invitrogen) and eluted in 1 ml fractions with 3 mM desthiobiotin (Sigma-Aldrich) in PBS. Protein concentration was determined by absorbance at 280 nm. Aliquots were frozen and stored at −80 degrees Celsius. Soluble Eph-Fc and Ephrin-Fc fusion proteins were purchased from R&D Systems.

### Virus and entry assays

RRV-GFP 26-95 [48] was grown on primary rhesus fibroblasts. A confluent T175 flask (Corning) was inoculated with app. 50 000 PFU RRV-GFP 26-95, resulting in a multiplicity of infection below 0.001. Virus was allowed to replicate until the cell lawn was completely destroyed (approximately three weeks). The cell culture supernatant was cleared by centrifugation for 10 min at 3000 g and further stored at 4 degrees Celsius. A filtration step was left out as we observed a relatively strong retention of virus in some 0.45 µM filters and found the supernatant to be free of visible debris or GFP containing particles after centrifugation. The virus stock was diluted 1∶5 in cell culture medium for infection, and was tested on 293T cells consistently yielding around 20% green cells two days post infection. We did not observe a noticeable drop in virus titer over approximately 6 months. RRV-YFP 26-95 (to be described in detail in another manuscript) encodes an YFP expression cassette at the same locus as the GFP cassette in RRV-GFP and is derived from the RRV 26-95 BAC [49]. For high titer infection, virus was concentrated in a JA20 fixed angle rotor for 2 h at 50 000 g.

KSHV.219 was prepared from iSLK.219 cells [50]. The cells were induced with 1 µg/ml doxycyclin (Sigma-Aldrich) and kept in this medium for one week. Supernatant was then collected, cleared by centrifugation for 10 min at 3000 g, filtered through a 0.45 µm PES vacuum filter (Millipore) and stored at 4 degrees Celsius. The virus stock was diluted 1∶5 in cell culture medium and was tested on 293T cells and consistently yielded around 20–50% infected cells after two days. We did not observe a noticeable drop in virus titer over approximately 4 months.

For blocking experiments, a functional MOI in the range of 0.1 was targeted where possible as this allows for enough dynamic range without over-saturating the entry assay. Infection in that range was achieved by using our virus stock at 1∶5 dilution. Entry was quantified by flow cytometry on day two post infection unless otherwise stated, with sample sizes of 10 000 cells or more. Where indicated, a commercial LIVE/DEAD fixable far red (Invitrogen) exclusion dye viability assay was included.

Infection of BJAB cells with rKSHV.219 was achieved by inducing iSLK.219 cells with 2.5 µg/ml doxycyclin in 200 µl DMEM with 10% FBS in a 48 well plate. The general protocol was taken from Myoung et al. [7] with slight modification. BJAB cells were added after one day in 200 µl RPMI with 10% FBS. After the desired length of co-culture, the cells were harvested by vigorous pipetting in PBS, fixed with 2% paraformaldehyde (Santa Cruz) and stained with anti-CD20 clone L27 (10 µg/ml) (BectonDickenson) followed by anti-mouse-Cy5 (Southern Biotec), all in 10% goat serum in PBS (Invitrogen). The cells were then analyzed by flow cytometry. In a first gating step, the B cells were separated by FSC/SSC analysis from the iSLK.219 cells. This step was followed by another gate for Cy5-high CD20-positive cells. This CD20-positive population was then analyzed for expression of the GFP reporter gene. As additional controls, BJAB were incubated with non-induced iSLK.219 cells, and induced iSLK.219 cells cultured without BJAB cells were analyzed.

Lentiviruses pseudotyped with VSV-G and A-MLV glycoprotein were produced as described elsewhere [51]. Briefly, SIVmac239 encoding a GFP expression cassette instead of the nef gene and deleted in the env locus was transfected into 293T cells together with the respective glycoprotein expression-plasmid to produce infectious virus. The A-MLV expression plasmid was a kind gift from Michael Farzan, Harvard Medical School.

All experiments were performed as biological replicates with the number of replicates as indicated (n = 2, 3, or 4). Error bars always indicate standard deviation (sd) or range for n = 2.

### Affinity-purification of gH/gL-interacting proteins

Soluble gH-FcStrep/gL constructs from RRV 26-95 and 17577 and KSHV as well as FcStrep as a control were expressed in 293T cells. 15 ml of protein-containing supernatant were coupled to Streptactin beads (Qiagen) for 4 h. The beads were then washed and incubated with the lysate of 293T cells (app. 10^9^ cells per sample in 10 ml). Lysates were prepared in 1% NP40 150 mM NaCl 20 mM HEPES 1 mM EDTA with protease inhibitor cocktail (Roche) and cleared by centrifugation for 1 h at 20000 g in 1.5 ml tubes. After overnight incubation with the lysate, the Streptactin beads were washed twice briefly with 1% NP40 in PBS and then eluted with 3 mM Desthiobiotin (Sigma-Aldrich) in 0.375% NP40 in PBS. Eluates were re-precipitated with ProteinG sepharose beads (GE Healthcare) for 1 h. The ProteinG beads were then washed briefly 3× with 1% NP40 in PBS, heated in SDS-sample buffer and subjected to electrophoresis on 8–16% gradient Laemmli gels (Invitrogen). Gels were stained with either colloidal coomassie SafeStain (Invitrogen) or SilverQest silver staining kit (Invitrogen). Visible bands were excised and sent to Taplin Mass Spectrometry Core Facility at Harvard Medical School for tryptic digest, LC-MS/MS analysis and database search.

### Flow cytometry based binding assay

293T cells were transfected with the respective Eph constructs. Two days after transfection, the cells were harvested without trypsinization in cold PBS and fixed for 10 min in 2% paraformaldehyde in PBS on ice, followed by 5 min of permeabilization in 0.1% NP40 in PBS. The cells were then blocked for 2 h in binding buffer (10% FBS in PBS) and incubated with the indicated Fc-fusion proteins at a concentration of approximately 10 nM (3 µg/ml as determined by A280 for gH-Fc/gL fusions) in binding buffer for 1 h. The cells were then washed in 30× the original volume in binding buffer for 1 h and then incubated with goat anti-human-Alexa488 (Invitrogen) 1∶100 and goat anti-mouse-Cy5 (Southern Biotech) secondary antibody 1∶100 for 1 h, followed by two washes for 30 min in 30× the original volume in PBS. The cells were then post-fixated and stored in 100 µl 2% paraformaldehyde in PBS until analysis on a FACScalibur (BectonDickinson). Flow cytometry files were analyzed using FloJo Version 8.8.7 (Tree Star).

### Isolation of nuclei and real-time PCR on viral genomes

HUVEC cells and rhesus fibroblasts were infected with RRV-YFP 26-95 (approximately 10^8^ viral genome copies in 800 µl in a sixwell plate and conditions that usually yield 70–95% infected rhesus fibroblasts or HUVEC, respectively) for 4 h. Virus was removed, the cells were washed 2× with PBS and harvested in 900 µl 0.05% Trypsin/EDTA solution (Invitrogen). Trypsin was stopped by addition of 100 µl FBS and the cells were pelleted for 5 min at 500 g in a microcentrifuge tube. The cell pellet was resuspended by vortexing in PBS and pelleted again. Nuclei were isolated in Nuclei EZ Prep buffer (Sigma-Aldrich) according to the manufacturer's recommendations. All isolation steps were carried out on ice and with a pre-chilled centrifuge. Briefly, 150 µl Nuclei EZ Prep buffer (Sigma-Aldrich) were added and the pellet was resuspended by 2 s of vortexing, followed by addition of 1 ml of Nuclei EZ Prep buffer and 1 s of vortexing. The cells were then incubated on ice for 10 min. Nuclei were pelleted at 500 g for 5 min and the supernatant was discarded. Resuspension, vortexing, and incubation in Nuclei EZ Prep buffer was repeated as above and the nuclei were pelleted again, discarding the supernatant. As an additional purification step, the nuclei pellet was resuspended in 1 ml of 6% iodixanol in PBS by brief vortexing. The nuclei were re-pelleted at 20000 g for 30 s. The supernatant was discarded and the nuclei were stored at −20C for analysis. Total nucleic acid was extracted using the Qiaamp (Qiagen) kit. Genomic viral DNA (LANA locus; forward primer: ACCGCCTGTTGCGTGTTA, reverse primer: CAATCGCCAACGCCTCAA, probe: CAGGCCCCATCCCC) and the cellular GAPDH locus (assay ID Hs02786624_g1, Applied Biosystems; primers and probe recognize both human and rhesus) were quantified by Taqman Realtime PCR using a LANA containing cosmid as RRV standard and serially diluted genomic cellular DNA as GAPDH standard. Three independent experiments were performed. Realtime PCR quantification was performed in duplicates. Results were normalized to the average LANA/genomic DNA ratio in each cell type with control protein (EGFR-Fc) set to 100%.

## Supporting Information

Figure S1 **Gating strategy for flow cytometry binding assay.** 293T cells were transfected with expression plasmids for myc epitope-tagged Eph proteins. The cells were fixed and permeabilized and incubated with anti-myc monoclonal antibody and either gH-Fc/gL or Fc control protein. Myc-antibody or bound Fc-fusion protein was detected with anti-mouse-Cy5 and anti-human-Alexa488 secondary antibodies. Exemplarily, cells transfected with empty vector, EphA2 or EphB4 expression plasmids are shown after incubation with either Fc or KSHV gH-Fc/gL. In each zebra plot, the coloration represents the density of events. The geometric mean of the intensities for Alexa488 and Cy5 in the area of analysis was determined and the ratio calculated.
(TIF)

Figure S2 **Binding preferences of A-type Ephrins for EphA2, EphA4 and EphA5.** 293T cells were transfected with the indicated Eph-constructs (myc-tag). The cells were lysed and equal amounts of lysate were immunoprecipitated with 1 µg of the recombinant Ephrin-Fc proteins. After washing three times with lysis buffer, bound protein was detected by Western Blot analysis.
(TIF)

Figure S3 **Effects of MOI or time of measurement on apparent RRV entry into cells.** (**A**) Rhesus fibroblasts and HUVEC were infected with RRV-YFP 26-95 at different MOIs. The viral inoculum was pre-incubated with EphB3-Fc (black boxes, solid line) or EGFR-Fc (black diamonds, dashed line) at 10 µg/ml for 45 min. Entry was quantified by flow cytometry two days after infection as number of YFP-positive cells. (n = 2, error bar represents range; if not visible, range is smaller than chart symbol) (**B**) The geometric mean fluorescence intensity of the YFP reporter gene was quantified from the same samples as in (B). (**C**) Percent inhibition achieved at different MOIs based on the percentage of green rhesus fibroblasts (black triangles, solid line) or HUVEC (black circles, dotted line). (**D**) Fold reduction in YFP fluorescence based on GFP fluorescence measured in rhesus fibroblasts (black triangles, solid line) or HUVEC (black circles, dotted line). (**E**) Rhesus fibroblasts and HUVEC were infected with RRV-GFP 26-95 which was pre-incubated with increasing concentrations of EphB3-Fc (black boxes, solid line) or Fc (control, black diamonds, dashed line). Entry was quantified as the number of GFP positive cells by flow cytometry 24 h post infection. (n = 2, error bar represents range)
(TIF)

Table S1 **Table of NCBI database accession numbers for Eph DNA sequences, protein sequences, and percentages of amino acid identity with rhesus monkey and mouse orthologs.**
(PDF)
