# Supplementary figures and images for: Rhesus Monkey Rhadinovirus Uses Eph Family Receptors for Entry into B Cells and Endothelial Cells but Not Fibroblasts
Source: PLoS Pathog. 2013 May 16;9(5):e1003360. doi: 10.1371/journal.ppat.1003360 (PMC3656109; doi:10.1371/journal.ppat.1003360)

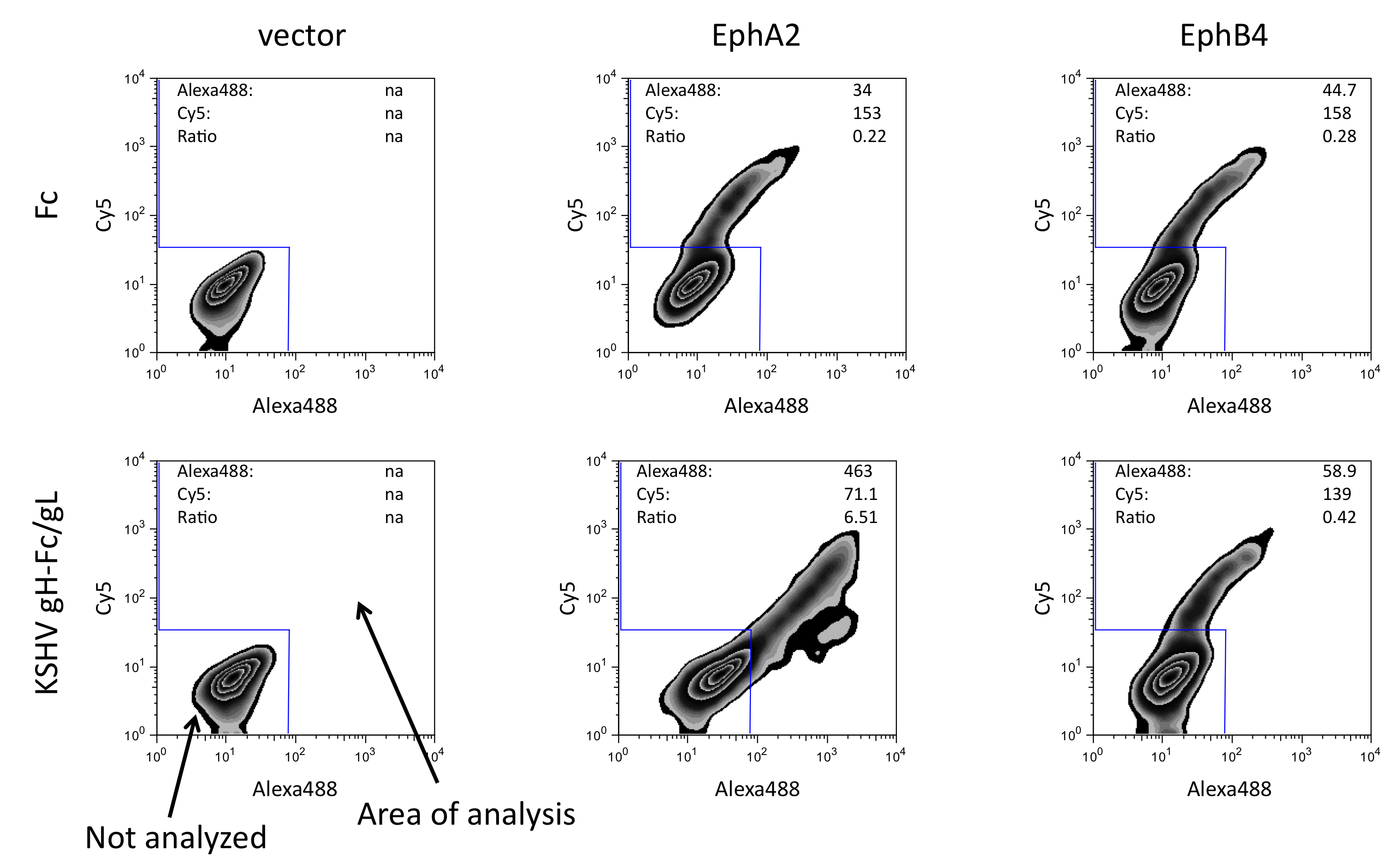

Supplement: Figure S1 — Gating strategy for flow cytometry binding assay. 293T cells were transfected with expression plasmids for myc epitope-tagged Eph proteins. The cells were fixed and permeabilized and incubated with anti-myc monoclonal antibody and either gH-Fc/gL or Fc control protein. Myc-antibody or bound Fc-fusion protein was detected with anti-mouse-Cy5 and anti-human-Alexa488 secondary antibodies. Exemplarily, cells transfected with empty vector, EphA2 or EphB4 expression plasmids are shown after incubation with either Fc or KSHV gH-Fc/gL. In each zebra plot, the coloration represents the density of events. The geometric mean of the intensities for Alexa488 and Cy5 in the area of analysis was determined and the ratio calculated. (TIF) [file ppat.1003360.s001.tif]

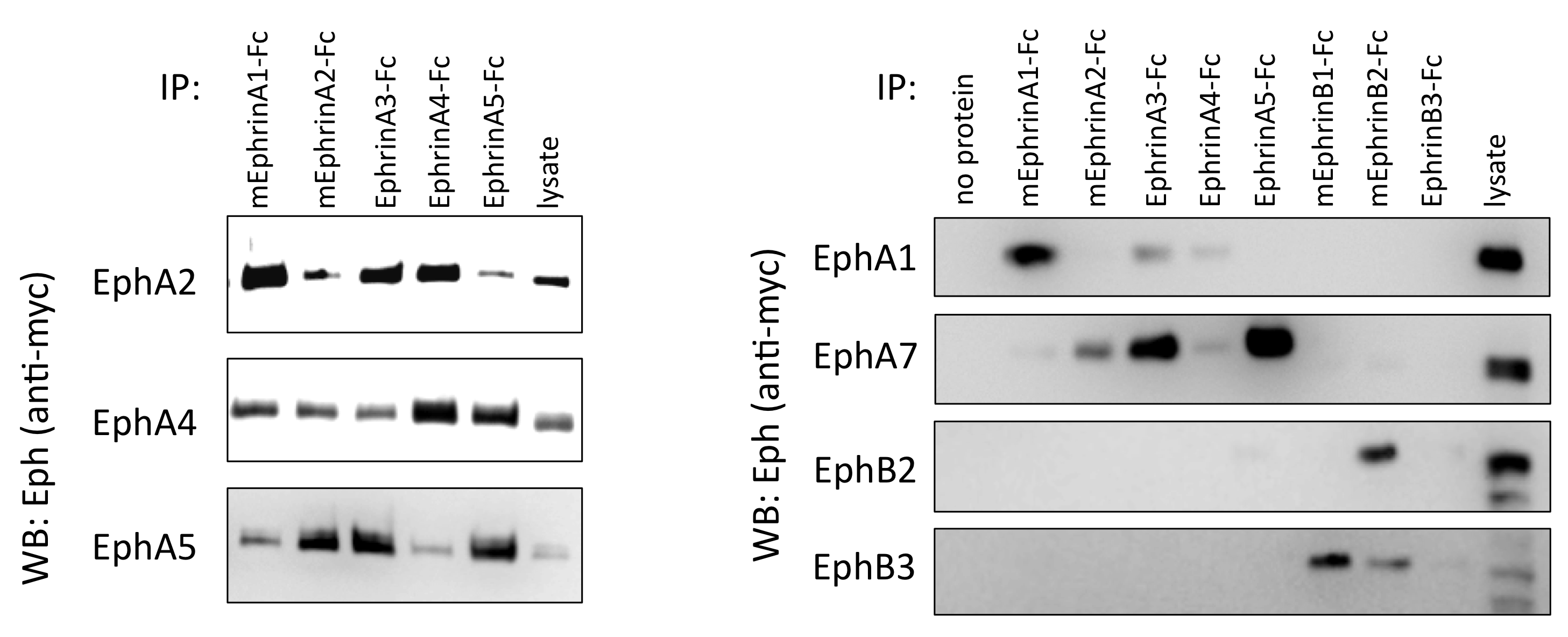

Supplement: Figure S2 — Binding preferences of A-type Ephrins for EphA2, EphA4 and EphA5. 293T cells were transfected with the indicated Eph-constructs (myc-tag). The cells were lysed and equal amounts of lysate were immunoprecipitated with 1 µg of the recombinant Ephrin-Fc proteins. After washing three times with lysis buffer, bound protein was detected by Western Blot analysis. (TIF) [file ppat.1003360.s002.tif]

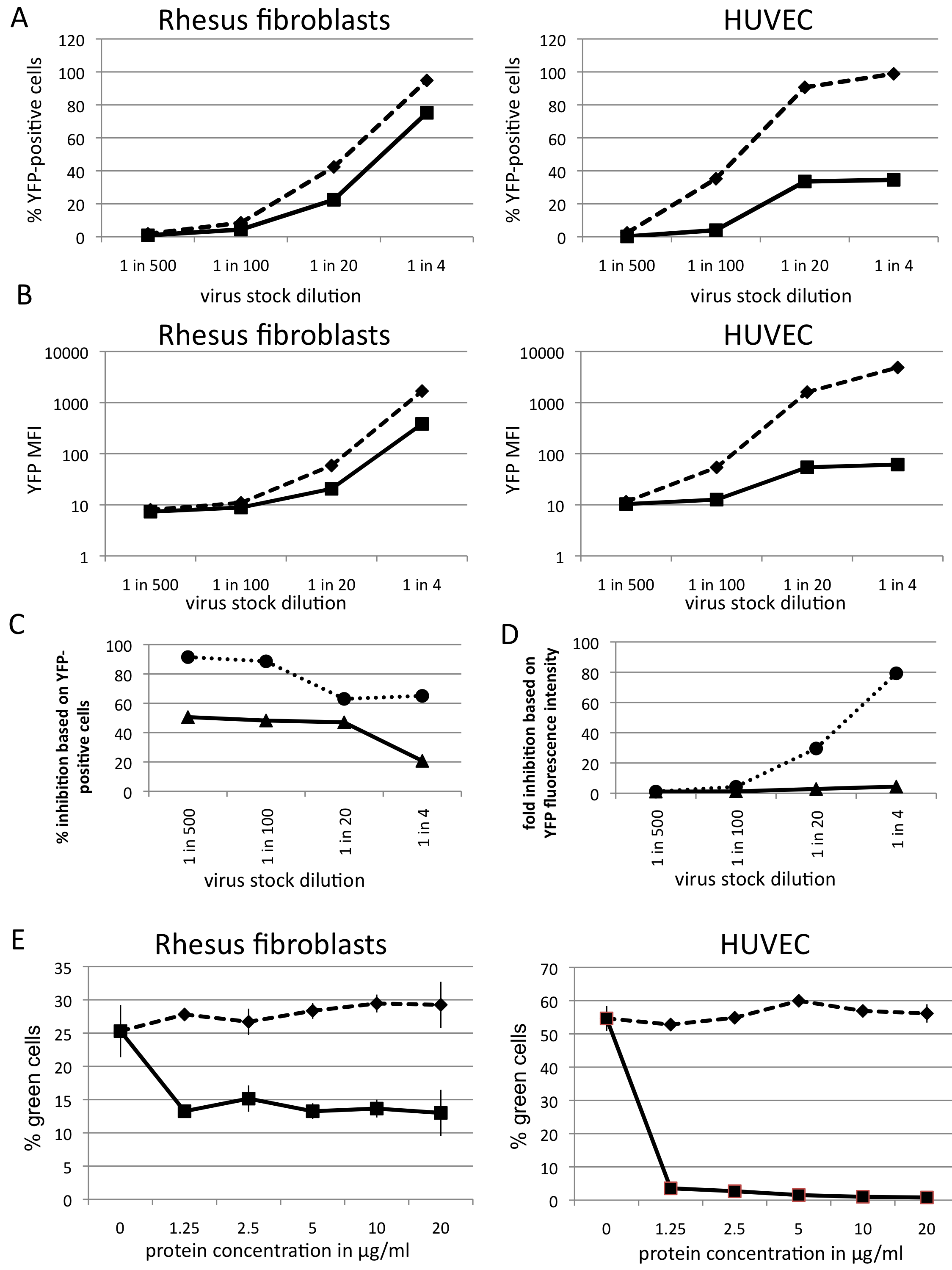

Supplement: Figure S3 — Effects of MOI or time of measurement on apparent RRV entry into cells. (A) Rhesus fibroblasts and HUVEC were infected with RRV-YFP 26-95 at different MOIs. The viral inoculum was pre-incubated with EphB3-Fc (black boxes, solid line) or EGFR-Fc (black diamonds, dashed line) at 10 µg/ml for 45 min. Entry was quantified by flow cytometry two days after infection as number of YFP-positive cells. (n = 2, error bar represents range; if not visible, range is smaller than chart symbol) (B) The geometric mean fluorescence intensity of the YFP reporter gene was quantified from the same samples as in (B). (C) Percent inhibition achieved at different MOIs based on the percentage of green rhesus fibroblasts (black triangles, solid line) or HUVEC (black circles, dotted line). (D) Fold reduction in YFP fluorescence based on GFP fluorescence measured in rhesus fibroblasts (black triangles, solid line) or HUVEC (black circles, dotted line). (E) Rhesus fibroblasts and HUVEC were infected with RRV-GFP 26-95 which was pre-incubated with increasing concentrations of EphB3-Fc (black boxes, solid line) or Fc (control, black diamonds, dashed line). Entry was quantified as the number of GFP positive cells by flow cytometry 24 h post infection. (n = 2, error bar represents range) (TIF) [file ppat.1003360.s003.tif]
