# Supplementary material for: Rhesus Monkey Rhadinovirus Uses Eph Family Receptors for Entry into B Cells and Endothelial Cells but Not Fibroblasts
Source: PLoS Pathog. 2013 May 16;9(5):e1003360. doi: 10.1371/journal.ppat.1003360 (PMC3656109; doi:10.1371/journal.ppat.1003360)
Supplement: Table S1 — Table of NCBI database accession numbers for Eph DNA sequences, protein sequences, and percentages of amino acid identity with rhesus monkey and mouse orthologs. (PDF) [file ppat.1003360.s004.pdf]

Eph	NCBI sequence accession number	NCBI protein sequence accession number	Amino acid sequence identity with rhesus monkey	Amino acid sequence identity with mouse
EphA1	BC130291	AAI30292	97%	88%
EphA2	NM_004431	P29317	98%	92%
EphA3	BC063282	AAH63282	99%	96%
EphA4	NM_004438	NP_004429	99%	99%
EphA5	NM_004439	NP_004430	99%	96%
EphA6	NM_001080448	NP_001073917	99%	95%
EphA7	BC143857	AAI43858	98%	98%
EphA8	BC141436	AAI41437	99%	95%
EphA10	NM_001099439	NP_001092909	96% (hypothetical sequence only)	93%
EphB1	BC111744	AAI11745	99%	99%
EphB2	NM_017449	NP_059145	99%	99%
EphB3	BC052968	NP_004434	99%	97%
EphB4	BC052804	AAH52804	99%	93%
EphB6	NM_004445	NP_004436	99%	92%
